# Supplementary motor area syndrome in intracranial gliomas is not associated with worse survival

**DOI:** 10.1007/s11060-026-05805-y

**Published:** 2026-09-23

**Authors:** Dragan Jankovic, Sophie Rosenke, Jens Blobner, Andrea Szelényi, Veit Stöcklein, Santhosh G. Thavarajasingam, Andreas Kramer, Philipp Karschnia, Nico Teske, Florian Ringel, Darius Kalasauskas

**Affiliations:** 1 Department of Neurosurgery, LMU University Hospital, LMU Medizin, Ludwig- Maximilians-Universität, Munich, Germany; 2 Faculty of Medicine, Lancaster University, Lancaster, UK; 3 Department of Neurosurgery, Technical University Munich, Munich, Germany; 4 Department of Neurosurgery, Uniklinikum Erlangen, Friedrich-Alexander-University Erlangen-Nuremberg, Erlangen, Germany

**Keywords:** Supplementary motor area, Glioma, SMA syndrome, Functional outcome, Extent of resection

## Abstract

**Purpose:**

Resection of gliomas involving the supplementary motor area (SMA) carries a substantial risk of postoperative functional deficits, which may affect patients’ ability to undergo adjuvant therapy. This study evaluated whether postoperative SMA syndrome is associated with delayed initiation of adjuvant therapy and with progression-free survival (PFS) and overall survival (OS).

**Methods:**

We performed a retrospective single-center study of patients undergoing resection of gliomas involving, adjacent to, or approached through the SMA between 2015 and 2024. SMA syndrome was defined as new-onset motor deficits and/or language impairment without corresponding intraoperative neuromonitoring changes and without involvement of the primary motor cortex or corticospinal tract. Logistic and Cox regression analyses were performed.

**Results:**

Seventy-four patients were included, of whom 37 (50%) developed postoperative SMA syndrome. In univariate logistic regression, RANO class 2 A and class 2B resections were associated with higher odds of postoperative SMA syndrome compared with RANO class 1 resections (*p* = 0.048 and *p* = 0.035, respectively). In the pairwise multivariable model, grouped RANO class 2 resections remained associated with higher odds of postoperative SMA syndrome compared with class 1 resections (OR 5.05, 95% CI 1.24–20.57; *p* = 0.024). Postoperative SMA syndrome was not associated with delayed initiation of adjuvant therapy (HR 0.86, 95% CI 0.49–1.53; *p* = 0.617), PFS (HR 1.21, 95% CI 0.63–2.32; *p* = 0.573), or OS (HR 1.21, 95% CI 0.49–2.95; *p* = 0.681). Postoperative functional decline (≥ 20-point KPS decrease) was independently associated with receipt of modified or reduced-intensity adjuvant therapy (adjusted OR 13.91, 95% CI 2.21–87.64; *p* = 0.005), whereas SMA syndrome itself was not associated with treatment modification.

**Conclusions:**

Postoperative SMA syndrome was not associated with delayed adjuvant therapy or worse survival. Functional status, rather than SMA syndrome, influenced treatment intensity, supporting maximal safe resection in the SMA region.

**Supplementary Information:**

The online version contains supplementary material available at https://doi.org/10.1007/s11060-026-05805-y.

## Introduction

Gliomas represent the most common primary malignant brain tumors and remain associated with poor prognosis, particularly in high-grade disease [1–3]. Current classification integrates histopathological and molecular features, distinguishing less-aggressive lower-grade IDH-mutant gliomas from IDH-wildtype glioblastoma the latter characterized by aggressive clinical behavior and limited survival despite multimodal therapy [2, 4, 5]. 

Maximal safe surgical resection is the gold standard of glioma management and has been repeatedly shown to improve progression-free and overall survival [6–9]. However, the infiltrative nature of diffuse gliomas may frequently limit the extent resection in eloquent cortical regions [10]. The supplementary motor area (SMA) anatomically defined as the cortical region located anterior to the primary motor cortex and rostral to the premotor cortex, is commonly involved in frontal gliomas and plays a critical role in motor initiation, coordination, and speech.

Resection within the SMA region carries a risk of postoperative neurological deficits, most notably SMA syndrome. It is characterised by contralateral hemiplegia or hemiparesis or hypokinesia, impaired motor initiation, speech disturbances such as mutism or reduced verbal fluency, and a general reduction in spontaneous activity [11]. Although typically transient, recovery is variable, and deficits may persist in a subset of patients [12, 13]. The reported incidence of SMA syndrome reaches 60.7% with glioma resections involving the SMA, but the overall incidence with all tumor types is variable (23%-100%) [12, 14]. 

Functional status represents a key prognostic factor in glioma patients and is consistently associated with survival across clinical studies, as it may affect influence the ability to tolerate adjuvant therapy and meet eligibility criteria for standard adjuvant therapy protocols [15–17]. Timely administration of adjuvant radiotherapy and chemotherapy is considered an essential component of care, particularly for high-grade tumors, and delays or treatment modifications have been associated with inferior outcomes [18, 19]. 

Despite the well-described clinical features and recovery patterns of SMA syndrome, its impact on oncological management remains unclear [20–23]. 

The aim of this study was therefore to identify risk factors for postoperative SMA syndrome and to determine whether it is associated with delayed initiation or modification of adjuvant therapy, and whether it affects progression-free or overall survival.

## Materials and methods

### Study design and population

This retrospective single-center study included consecutive patients with intracranial gliomas who underwent surgical resection between 2015 and 2024. All data were anonymized prior to analysis. The study was conducted in accordance with the Declaration of Helsinki and is reported following the STROBE guidelines for observational studies. Ethical approval was obtained from the local ethics committee (LMU Munich, No. 25–0709).

**Fig. 1:**
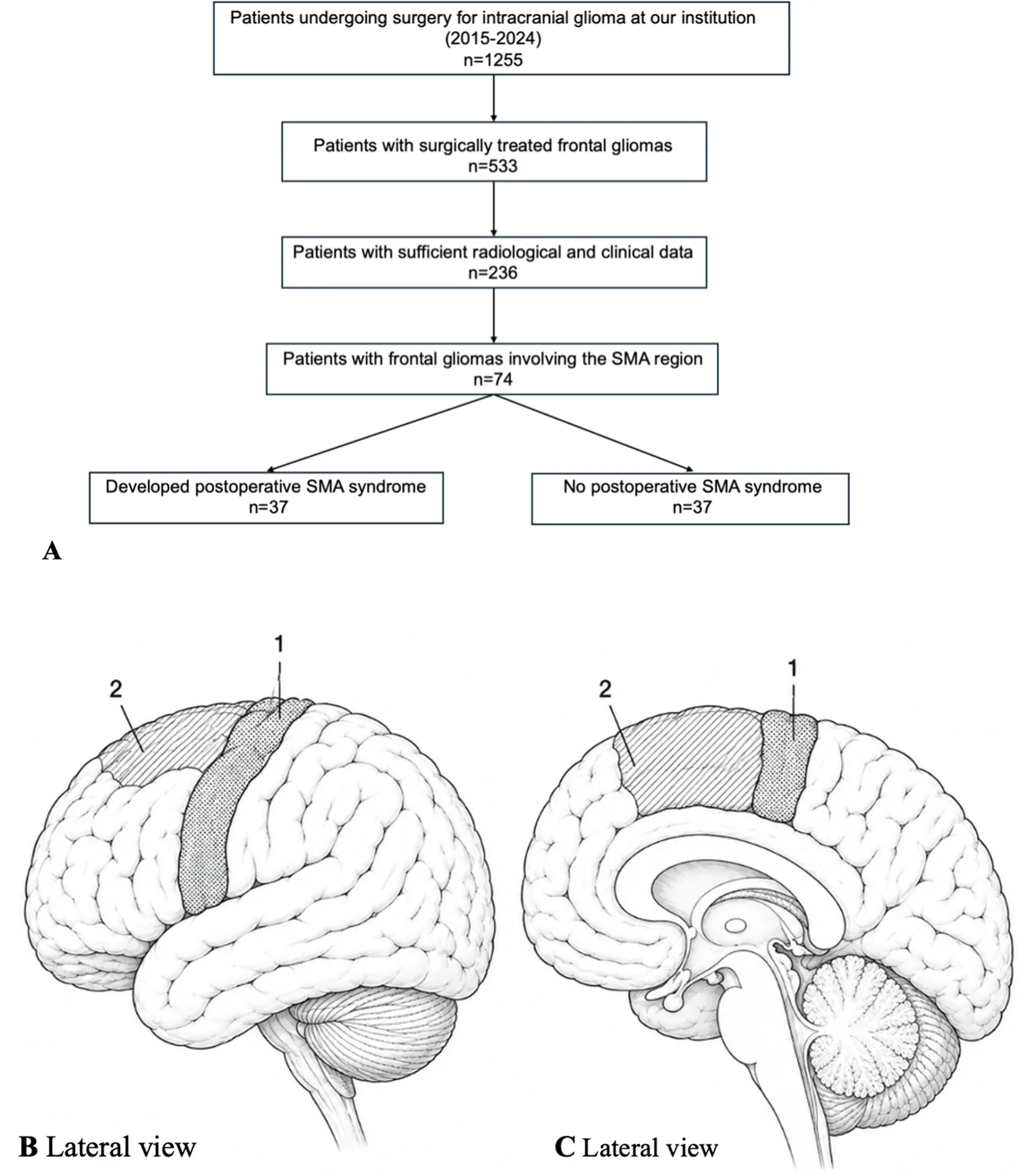
Flowchart of patient selection for the study cohort (**A**); Anatomical delineation of the supplementary motor area (SMA). The lateral (**1B**) and medial (**1C**) views of the cerebral hemisphere are shown. The SMA is highlighted in relation to adjacent motor areas. Regions depicted are: (1) precentral gyrus (2), supplementary motor cortex (SMA)

Patients with histopathologically confirmed CNS WHO grade 2–4 gliomas involving the SMA, located in its immediate vicinity, or requiring a surgical approach through the SMA were included. A flowchart of patient selection and study cohort formation is shown in Fig. 1A. The SMA was anatomically defined by the superior frontal sulcus laterally, the interhemispheric fissure medially, and the cingulate sulcus inferiorly, and extending from the precentral sulcus to approximately 5 cm anteriorly. **(**Fig. 1B-C**)** [24, 25] Inclusion required availability of complete clinical, imaging, and follow-up data. Patients undergoing biopsy only, without SMA involvement or proximity, or with incomplete data were excluded.

### Clinical and functional assessment

Demographic and clinical data included sex, age at the time of diagnosis, neurological and functional status before and after surgery. Functional status was assessed longitudinally using the Karnofsky Performance Status (KPS) preoperatively, postoperatively, at discharge, and at 3-month follow-up [26]. Discharge disposition was categorized into three groups: discharge to home, discharge to a rehabilitation facility, or transfer to another hospital department for further inpatient treatment.

The postoperative SMA syndrome was recorded based on clinical documentation and defined as new-onset motor deficits and/or language impairment without corresponding changes in intraoperative neurophysiological monitoring and without radiological or surgical evidence of primary motor cortex or corticospinal tract injury or ischemia.

All surgical procedures were performed under general anesthesia. Intraoperative neuromonitoring (IONM) reports were systematically reviewed, including subcortical, cortical, and transcranial stimulation modalities. Changes in IONM signals were analyzed in relation to postoperative neurological status and imaging findings.

### Imaging and resection assessment

Preoperative MRI was reviewed for tumor characteristics, including location, midline shift, involvement of the precentral gyrus, infiltration of the corpus callosum, and contralateral involvement. Tumor focality was classified as unifocal (single lesion), multifocal or multicentric [27, 28]. A postoperative MRI performed within 72 h after surgery was used to determine the extent of resection (EOR). Tumors were classified according to the WHO 2021 classification [4]. For high-grade gliomas, EOR was assessed based on the residual contrast-enhancing (CE) tumor volume according to the RANO resection classification. RANO class 1 was defined as supramaximal CE resection (0 cm³ CE and ≤ 5 cm³ non-contrast-enhancing tumor), class 2 comprised complete (class 2 A; 0 cm³ CE with > 5 cm³ non-contrast-enhancing tumor) and near-total CE resection (class 2B; ≤1 cm³ residual CE tumor), whereas class 3 comprised subtotal (class 3 A; ≤5 cm³ residual CE tumor) and partial CE resection (class 3B; >5 cm³ residual CE tumor) [29]. 

For low-grade gliomas, EOR was assessed based on the residual T2-FLAIR hyperintense tumor volume according to the RANO classification. RANO class 1 corresponded to supramaximal T2-FLAIR resection (no residual T2-FLAIR tumor with resection beyond imaging abnormalities), class 2 to maximal resection (0–5 cm³ residual T2-FLAIR tumor), class 3 to submaximal resection (> 5–25 cm³ residual T2-FLAIR tumor), and class 4 to minimal resection (> 25 cm³ residual T2-FLAIR tumor and/or residual contrast-enhancing tumor) [30]. For the purposes of regression analyses, RANO classes 2 A and 2B (and, where applicable, 3 A and 3B) were pooled into single class 2 and class 3 categories due to limited subgroup sample sizes.

Three-dimensional volumetric assessment was performed using Brainlab Elements software (Brainlab AG, Munich, Germany) on preoperative and postoperative MRI scans. Preoperative tumor volume and postoperative residual tumor volume were determined by three-dimensional segmentation on consecutive MRI slices. For high-grade gliomas, contrast-enhancing tumor volume was assessed on contrast-enhanced T1-weighted images, whereas for low-grade gliomas, T2-FLAIR hyperintense tumor volume was assessed. Postoperative residual tumor volume was assessed on MRI obtained within 72 h after surgery. All volumetric assessments were independently reviewed by two board-certified neurosurgeons (D.J. and J.B.), with discrepancies resolved by consensus.

### Treatment parameters

Data on adjuvant therapy were obtained from medical records and tumor board protocols. Parameters included the time to initiation of adjuvant therapy, treatment modality (radiotherapy, chemotherapy, or combined treatment), and intensity. Therapy was classified as standard or modified/reduced based on deviations from established treatment protocols.

### Endpoints and statistical analysis

Progression-free survival (PFS) was defined as the time from surgery to radiographic progression according to RANO 2.0 criteria. Overall survival (OS) was defined as the time from surgery to death from any cause.

Continuous variables are reported as median (IQR) and categorical variables as counts (%). Group comparisons between patients with and without postoperative SMA syndrome were performed using the Mann–Whitney U test for continuous variables and the chi-square test or Fisher’s exact test, as appropriate, for categorical variables. Logistic regression was used to identify factors associated with postoperative SMA syndrome.

For the multivariable logistic regression on SMA syndrome, age at operation, preoperative KPS, and tumor volume were included as standard clinical baseline covariates rather than mechanistic risk factors specific to SMA syndrome. The same covariates were retained as adjustment variables in the multivariable Cox models for time to adjuvant therapy, PFS, and OS, where they represent established prognostic factors in glioma cohorts.

A sensitivity analysis was performed by replacing SMA zone involvement with SMA border involvement to assess the robustness of the primary model.

Cox proportional hazards models were applied to assess associations with time to adjuvant therapy, PFS, and OS, adjusted for age, preoperative KPS, and tumor size. Additional Cox regression models sequentially included delay to adjuvant therapy, postoperative KPS, follow-up KPS, extent of resection, MGMT status, and WHO grade. Survival curves were estimated using the Kaplan–Meier method, and differences between groups were assessed using the log-rank test. All analyses were conducted in R (Version 4.5.1 Foundation for Statistical Computing, Vienna, Austria). A two-sided p-value ≤ 0.05 was considered statistically significant.

Model fit was assessed using Nagelkerke’s R² for logistic regression models and Harrell’s concordance index (C-index) for Cox proportional hazards models **(Supplementary Table 1)**.

## Results

### Patient demographics

A total of 74 patients were included, of whom 37 (50%) developed postoperative SMA syndrome (Fig. 1A). Baseline characteristics are summarized in Table 1. Median age at operation (53 vs. 61 years, *p* = 0.925), and sex distribution (males 59.5% vs. 45.9%, *p* = 0.352) was comparable between groups. Preoperative KPS was modestly lower among patients who developed SMA syndrome (median 90 vs. 80, *p* = 0.112). KPS at discharge was significantly lower in patients with postoperative SMA syndrome (median 80, IQR 70–80) compared with the non-SMA group (median 80, IQR 80–90; *p* = 0.006), although both groups had recovered to a median KPS of 90 by follow-up (*p* = 0.902). Precentral gyrus involvement, corpus callosum infiltration, and midline shift were more frequent in patients with SMA syndrome (5.4% vs. 16.2%, *p* = 0.261; 24.3% vs. 35.1%, *p* = 0.446; and 24.3% vs. 40.5%, *p* = 0.214, respectively), without reaching statistical significance. Postoperative infarction (without involvement of the primary motor cortex or corticospinal tract) was numerically more frequent in the SMA group (18.9% vs. 2.7% in the non-SMA group); however, this difference did not reach statistical significance (*p* = 0.056). Intraoperative neuromonitoring was used in 75.7% of patients in each group, without a significant difference in utilization between groups. IONM alterations were documented in four patients. In two cases, MEP deterioration resulted in limitation of further resection. One of these patients had a small postoperative infarction without a new neurological deficit, whereas the other developed postoperative hemiparesis without evidence of infarction. In the remaining two cases, transient MEP decrease or fluctuation occurred without limiting the extent of resection and without new postoperative neurological deficits.

Similarly, the distribution of extent of resection showed a numerical difference between groups but did not reach statistical significance (*p* = 0.054). RANO class 1 resection was more frequent in the non-SMA group (35.1% vs. 13.5%), whereas RANO class 2 resection was more frequent in patients with SMA syndrome (43.2% vs. 67.6%). RANO class 3 resection was comparable between groups (21.6% vs. 18.9%). Histopathological and molecular tumor characteristics were comparable between groups. No significant differences were observed regarding CNS WHO grade (*p* = 0.609), IDH mutation status (*p* = 0.352), 1p/19q codeletion status (*p* = 0.206), or MGMT promoter methylation status (*p* = 0.433).Similarly, the proportion of patients requiring adjuvant therapy following multidisciplinary tumor board recommendation was identical between groups (75.7% in both groups, *p* = 1.000), and the median time from surgery to initiation of adjuvant therapy was comparable (28.0 [IQR 21.5–39.5] vs. 27.0 [IQR 20.0–56.8] days, *p* = 0.836).

At first outpatient follow-up (median 43 days, IQR (15.5–97.5)), 7/37 (18.9%) still had deficits, while 30/37 (81.1%) had recovered, indicating that SMA syndrome was predominantly transient.

**Table 1:** Baseline characteristics

Variable	Non-SMA group (*n* = 37)	SMA group (*n* = 37)	*p*-value
Age at operation (years) (median [IQR])	53.50 [40.50, 65.50]	61.00 [33.75, 64.75]	0.925
Male sex, n (%)	22/37 (59.5%)	17/37 (45.9%)	0.352
KPS, preoperative	90 [80–100]	80 [70–90]	0.112
KPS at discharge	80 [80–90]	80 [70–80]	0.006
KPS at follow-up	90 [80–90]	90 [80–90]	0.902
Preoperative tumour volume, cm³, median [IQR]	**37.80 [15.70–55.40]**	**29.20 [20.40–70.10]**	**0.994**
Right hemispheric location, n (%)	24/37 (64.9%)	20/37 (54.1%)	0.478
Tumor involving SMA, n (%)	29/37 (78.4%)	33/37 (89.2%)	0.345
Tumor in immediate vicinity of SMA, n (%)	8/37 (21.6%)	2/37 (5.4%)	0.085
Surgical approach through SMA, n (%)	0/37 (0.0%)	2/37 (5.4%)	0.493
Unifocal tumor, n (%)	33/37 (89.2%)	33/37 (89.2%)	1.000
Midline shift, n (%)	9/37 (24.3%)	15/37 (40.5%)	0.214
Precentral gyrus involvement, n (%)	2/37 (5.4%)	6/37 (16.2%)	0.261
Corpus callosum involvement, n (%)	9/37 (24.3%)	13/37 (35.1%)	0.446
Speech area involvement, n (%)	2/37 (5.4%)	2/37 (5.4%)	1.000
Postoperative infarction, n (%)*	1/37 (2.7%)	7/37 (18.9%)	0.056
**Extent of resection** **(RANO classification), n (%)**			**0.054**
Class 1	13/37 (35.1%)	5/37 (13.5%)	
Class 2	16/37 (43.2%)	25/37 (67.6%)	
Class 3	8/37 (21.6%)	7/37 (18.9%)	
**CNS WHO Grade**,** n (%)**			0.609
Grade 2	9/37 (24.3%)	8/37 (21.6%)	
Grade 3	8/37 (21.6%)	5/37 (13.5%)	
Grade 4	20/37 (54.1%)	24/37 (64.9%)	
**IDH mutation status n (%)**			
IDH-mutated IDH-wildtype	20/37 (54.1%) 17/37 (45.9%)	15/37 (40.5%) 22/37(59.5%)	0.352
**1p/19q codeletion status**,** n (%)**	9/37 (24.3%)	6/37(16.2%)	0.206
**MGMT promoter methylation status n (%)**			
Unmethylated Methylated	8/37 (21.6%) 29/37(78.4%)	12/37 (32.4%) 25/37 (67.6%)	0.433
**Adjuvant therapy recommended by multidisciplinary tumor board**,** n (%)**	28/37 (75.7%)	28/37 (75.7%)	1.000
**Time from surgery to initiation of adjuvant therapy**,** days (median [IQR])**	28.0 [21.5–39.5]	27.0 [20.0–56.8]	0.836

* Postoperative infarctions did not involve the primary motor cortex or corticospinal tract

SMA, supplementary motor area; KPS, Karnofsky Performance Status; IQR, interquartile range; CNS WHO, Central Nervous System World Health Organization; RANO, Response Assessment in Neuro-Oncology; IDH, isocitrate dehydrogenase; MGMT, O6-methylguanine-DNA methyltransferase; 1p/19q, combined loss of chromosome arms 1p and 19q

### Risk factors for postoperative SMA syndrome

In univariate logistic regression, RANO class 2 A and class 2B resections were associated with higher odds of postoperative SMA syndrome compared with RANO class 1 resections (class 2 A: OR 3.47, 95% CI 1.01–11.86; *p* = 0.048; class 2B: OR 13.00, 95% CI 1.20-140.74; *p* = 0.035).

IONM-limited tumor resection showed a trend toward increased risk (OR 3.06, 95% CI 0.86–10.84; *p* = 0.084). No other variables reached statistical significance in univariate analysis (Table 2).

In the primary multivariable logistic regression model, including SMA zone involvement, age at operation, preoperative KPS, and tumor size, no variable was independently associated with postoperative SMA syndrome.

In the pairwise multivariable model, grouped RANO class 2 resections remained associated with higher odds of postoperative SMA syndrome compared with class 1 resections (OR 5.05, 95% CI 1.24–20.57; *p* = 0.024, Table 2).

SMA zone involvement was not independently associated with SMA syndrome after adjustment (OR 1.98, 95% CI 0.52–7.50; *p* = 0.313). A sensitivity analysis replacing SMA zone involvement with SMA border involvement yielded consistent findings, without materially altering the results of the primary model.

**Table 2:** Logistic regression analysis of predictors of postoperative SMA syndrome

Variable	Univariate OR (95% CI)	*p* value	Multivariable OR (95% CI)*	*p* value	Nagelkerke *R*^2^
Extent of resection (RANO classification)					
class 2 A†	3.47 (1.01–11.86)	0.048			
class 2B†	13.00 (1.20-140.74)	0.035			
class 3 A†	3.64 (0.78–17.03)	0.101			
class 2 vs. 1			5.05 (1.24–20.57)	0.024	0.071
class 3 vs. 2			0.60 (0.17–2.08)	0.418	0.208
Resection limited by IONM	3.06 (0.86–10.84)	0.084	2.22 (0.55–8.88)	0.260	0.093
Precentral gyrus involvement	3.39 (0.64–18.02)	0.153	3.30 (0.58–18.72)	0.182	0.106
SMA zone involvement (vs. vicinity/approach)	2.28 (0.62–8.35)	0.215	1.98 (0.52–7.50)	0.313	0.089
Age at operation (per year)	1.00 (0.97–1.03)	0.816	0.98 (0.95–1.02)	0.335	0.089
Preoperative KPS (per point)	0.98 (0.95–1.01)	0.182	0.97 (0.93–1.00)	0.085	0.089
Tumor size (cm³)	1.00 (0.99–1.01)	0.764	1.00 (0.98–1.01)	0.879	0.089

†RANO class 2 used as reference category. RANO class 3B excluded due to absence of SMA syndrome events

IONM = intraoperative neuromonitoring; SMA = supplementary motor area; KPS = Karnofsky Performance Status

### Time to initiation of adjuvant therapy

In univariate Cox proportional hazards analysis, lower preoperative Karnofsky Performance Status (KPS) was associated with delayed initiation of adjuvant therapy (HR 0.98, 95% CI 0.97–1.00; *p* = 0.026). Discharge destination was also associated with therapy timing, with delayed initiation in patients discharged to non-home settings (HR 2.17, 95% CI 1.19–3.95; *p* = 0.011), particularly in those transferred to other hospital departments (HR 3.15, 95% CI 1.56–6.36; *p* = 0.001). Higher WHO grade was associated with earlier therapy initiation (grade 3 vs. grade 2: HR 5.38, 95% CI 1.50–19.24; *p* = 0.010; grade 4 vs. grade 2: HR 6.62, 95% CI 2.02–21.67; *p* = 0.002). Age at operation, sex, tumor volume, RANO resection extent, and tumor board intent were not significantly associated with therapy timing in univariate analysis.

**Fig. 2:**
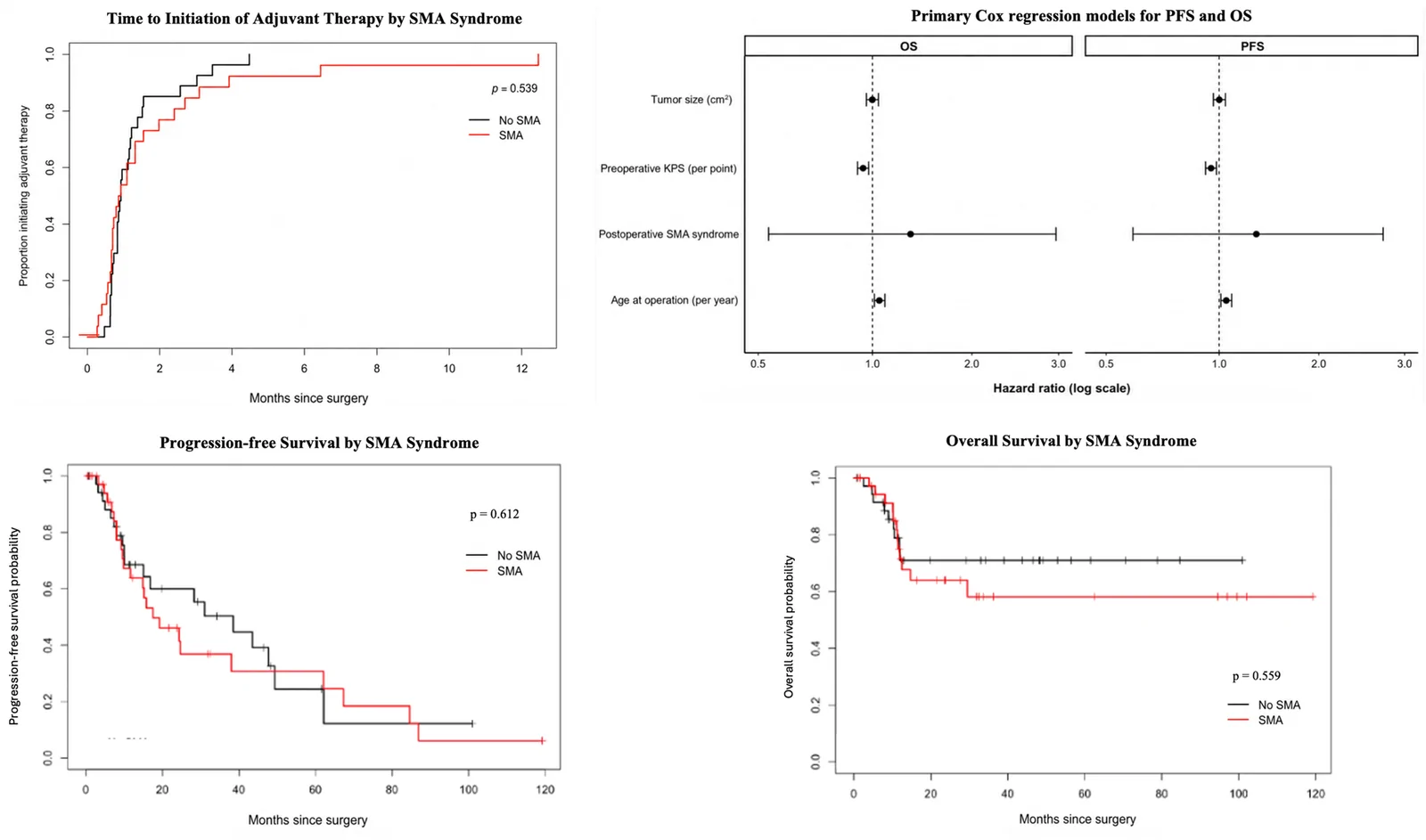
Association of postoperative supplementary motor area (SMA) syndrome with time to therapy initation, progression-free survival (PFS) and overall survival (OS). (**A**) Kaplan–Meier curve for time to therapy initiation by SMA status (**A**); (**B**) Multivariable Cox regression models for PFS and OS. (**C**) Kaplan–Meier estimate of PFS stratified by postoperative SMA syndrome status. (**D**) Kaplan–Meier estimate of OS stratified by postoperative SMA syndrome status. Cox regression models were adjusted for age at operation, preoperative Karnofsky Performance Status (KPS), and tumor size. Hazard ratios (HRs) are presented with 95% confidence intervals

In multivariable Cox proportional hazards regression adjusted for age at operation, preoperative KPS, and tumor volume, postoperative SMA syndrome was not associated with time to initiation of adjuvant therapy (HR 0.86, 95% CI 0.49–1.53; *p* = 0.617). Neither age at operation nor preoperative KPS remained independently associated with therapy timing in the adjusted model. Kaplan–Meier analysis demonstrated no statistically significant difference in time to therapy initiation between patients with and without postoperative SMA syndrome (*p* = 0.539; Fig. 2A).

### Survival outcomes

In univariate analysis, WHO grade 4 (vs. grade 2) was associated with worse progression-free survival (PFS) (HR 4.70, 95% CI 1.86–11.85; *p* = 0.001) and overall survival (OS) (HR 9.90, 95% CI 1.32–74.23; *p* = 0.026). MGMT promoter methylation was associated with improved PFS (HR 0.33, 95% CI 0.16–0.67; *p* = 0.002) and OS (HR 0.34, 95% CI 0.14–0.81; *p* = 0.015). Extent of resection was associated with improved OS (HR 0.27, 95% CI 0.11–0.67; *p* = 0.005).

In the multivariable Cox regression model, postoperative SMA syndrome was not associated with progression-free survival (HR 1.21, 95% CI 0.63–2.32; *p* = 0.573) or overall survival (HR 1.21, 95% CI 0.49–2.95; *p* = 0.681). Increasing age at operation was independently associated with poorer PFS (HR 1.04, 95% CI 1.02–1.06; *p* = 0.001) and OS (HR 1.04, 95% CI 1.01–1.07; *p* = 0.013), whereas preoperative as well as postoperative KPS and tumor size were not independently associated with survival outcomes (Fig. 2B).

Kaplan–Meier analyses confirmed no significant survival differences between patients with and without SMA syndrome (PFS log-rank *p* = 0.612; OS log-rank *p* = 0.559; Fig. 2C-D).

Persistent SMA syndrome was descriptively associated with shorter observed PFS compared with recovered SMA syndrome (median 9.2 vs. 15.1 months). However, univariate Cox regression did not demonstrate a statistically significant association with PFS (persistent vs. recovered SMA: HR 0.67, 95% CI 0.19–2.33; *p* = 0.529). Similarly, no significant difference in OS was observed between patients with persistent and recovered SMA syndrome (*p* = 0.145).

In an exploratory analysis, persistent SMA syndrome at follow-up was not associated with delayed therapy initiation (HR 0.91, 95% CI 0.32–2.60; *p* = 0.859) in a multivariable model adjusted for age at surgery, preoperative KPS, and tumor size (Supplementary Table 2). Given the small persistent-SMA subgroup (*n* = 7) and the wide confidence intervals, these exploratory findings should be interpreted cautiously.

### Functional outcomes and treatment intensity

Postoperative functional deterioration was significantly associated with modification of adjuvant treatment intensity. Among patients with a ≥ 20-point postoperative decline in KPS, 75% received non-standard or reduced-intensity therapy, compared with 30.4% among patients without significant KPS worsening (univariate OR 6.88, 95% CI 1.65–28.63; *p* = 0.008).

In an exploratory univariate Cox regression analysis, reduced/non-standard adjuvant treatment intensity was not significantly associated with PFS (HR 0.39, 95% CI 0.11–1.38; *p* = 0.143) or OS (HR 0.64, 95% CI 0.13–3.21; *p* = 0.588). In a separate analysis restricted to patients with CNS WHO grade 4 tumors, reduced/non-standard treatment intensity was likewise not significantly associated with PFS (HR 0.18, 95% CI 0.02–1.58; *p* = 0.122) or OS (HR 2.08, 95% CI 0.34–12.59; *p* = 0.425). Given the limited sample size and wide confidence intervals, particularly in the grade 4 subgroup, these findings should be interpreted as exploratory.

Postoperative SMA syndrome itself was not associated with treatment modification (OR 0.94, 95% CI 0.35–2.51; *p* = 0.906) (Table 3). After adjustment for postoperative SMA syndrome status, age at operation, preoperative KPS, and WHO grade, postoperative KPS worsening remained independently associated with modification of treatment intensity (adjusted OR 13.91, 95% CI 2.21–87.64; *p* = 0.005), suggesting that overall functional deterioration rather than SMA syndrome itself influenced oncological treatment decisions.

**Table 3:** Logistic regression analyses for receipt of non-standard adjuvant therapy

Variable	OR	95% CI	*p* value
Univariate model			
postoperative SMA syndrome	0.94	0.35–2.51	0.906
postoperative KPS worsening (≥ 20 points)	6.88	1.65–28.63	0.008
Multivariable model			
KPS worsening ≥ 20 points	13.91	2.21–87.64	0.005
Postoperative SMA syndrome	0.57	0.14–2.36	0.442
Age at operation (per year)	1.01	0.96–1.05	0.804
Preoperative KPS (per point)	0.97	0.93–1.02	0.251
WHO high grade (vs. low grade)	0.05	0.008–0.28	< 0.001

## Discussion

The present study investigated whether postoperative SMA syndrome adversely affects the oncological management in patients undergoing resection of SMA-associated gliomas. In this single-centre cohort, postoperative SMA syndrome was not associated with delayed initiation of adjuvant therapy, nor with reduced PFS or OS. Survival outcomes were instead determined by established biological prognostic factors, particularly patient age, WHO grade, MGMT methylation status, and extent of resection.

The observed survival difference between CNS WHO grade 2 and grade 4 tumors was expected given their distinct biological behavior and was not intended as a novel finding. Rather, the association of higher WHO grade with poorer survival provides internal validation that our heterogeneous cohort reproduces established prognostic relationships.

The 50% incidence of SMA syndrome in our cohort is consistent with rates reported in published glioma series. Young et al. reported the incidence of 60.7% in patients undergoing resection involving the SMA, with recovery strongly associated with integrity of the frontal aslant tract rather than the SMA itself [12]. A systematic review by Palmisciano demonstrated a wide range (23%–100%), reflecting heterogeneity in patient populations, surgical approaches and definitions [22]. Despite this high incidence, SMA syndrome did not emerge as a determinant of adjuvant therapy timing or survival in any analytical model.

De Maria and colleagues recently identified anatomical and surgical risk factors for postoperative neurological deficits in a bicentric SMA cohort, similarly highlighting the importance of resection extent and tumor location. Our analysis extends these findings by additionally evaluating the downstream effect of SMA syndrome on adjuvant treatment delivery and oncological outcomes — a dimension that, to our knowledge, has not been previously assessed.”

With regard to risk factors, resections classified as RANO 2 were associated with increased odds of postoperative SMA syndrome compared with supramaximal resection (RANO class 1). This finding likely reflects greater anatomical complexity and functional proximity of tumors in which maximal safe resection was constrained by eloquent motor regions. In our cohort, RANO class 2 resection frequently represented the limit of safe resection due to proximity to relevant functional structures, including the corticospinal tract or arcuate fasciculus. In addition, resection of SMA-associated gliomas may involve or place at risk the frontal aslant tract, which has been implicated in the occurrence and recovery of postoperative SMA syndrome [31]. 

Notably, intraoperative neuromonitoring (IONM) signal changes were not associated with SMA syndrome, supporting the concept that SMA syndrome arises from disruption of higher-order motor networks rather than injury to the corticospinal tract [32]. The trend toward increased SMA syndrome risk with IONM-limited resection (OR 3.06, 95% CI 0.86–10.84; *p* = 0.084) did not reach statistical significance, likely due to the limited number of patients in this subgroup (*n* = 14).

Tuncer et al. demonstrated that recovery from SMA syndrome in glioma patients is predicted by restoration of interhemispheric SMA connectivity through the corpus callosum — a pathway anatomically distinct from the corticospinal tract and not interrogated by standard monitoring [20]. In our cohort, postoperative SMA syndrome was predominantly transient. Recovery frequently extended beyond the immediate postoperative period but usually resolved up to the first out-patient follow-up. Among patients who did not recover by first follow-up, no significant differences in PFS or OS were observed.

Despite this relatively prolonged recovery period, postoperative SMA syndrome was not associated with delayed initiation of adjuvant therapy. Instead, discharge destination and baseline functional status appeared to be the principal determinants of therapy timing. This finding suggests that clinically meaningful early recovery, rather than complete neurological resolution, may be sufficient to permit initiation of adjuvant treatment within standard neuro-oncological timelines of approximately four to six weeks after surgery, consistent with prior reports [18, 19, 33]. 

Similarly, SMA syndrome was not associated with reduced PFS or OS. The absence of a survival disadvantage suggests that transient postoperative neurological deficits do not necessarily translate into impaired oncological outcomes. These findings support the principle of maximal safe resection, even in eloquent regions such as the SMA, as transient deficits alone should not preclude aggressive surgical strategies when oncologically indicated [2, 29, 30]. However, postoperative functional decline —independent of SMA syndrome — was significantly associated with reduced treatment intensity. Patients experiencing substantial deterioration in KPS were more likely to receive modified or less intensive therapy. This highlights that global functional impairment, rather than the specific occurrence of SMA syndrome, is the clinically relevant factor influencing treatment decisions. Importantly, a decline in KPS does not necessarily imply the occurrence of a new focal neurological deficit. As a global measure of functional status, postoperative KPS may be influenced by several factors, including frailty, delayed postoperative recovery, reduced mobility or independence, and systemic or postoperative complications such as pneumonia. Thus, the observed decline in KPS should be interpreted as a measure of overall postoperative functional deterioration rather than as a surrogate for a new focal neurological deficit.

The strong association between postoperative KPS decline and receipt of non-standard adjuvant therapy likely reflects multiple converging factors such as eligibility thresholds for full-intensity radiochemotherapy protocols, which often require KPS ≥ 70, tumor board decisions to modify therapy intensity in patients perceived as unable to tolerate standard regimens as well as patient and family preferences regarding aggressive treatment in the setting of postoperative deterioration. The fact that this association persisted independent of SMA syndrome status indicates that decision-makers calibrate adjuvant treatment to global functional reserve rather than to the specific neurological phenotype.

### Limitations

Several limitations should be acknowledged. The retrospective identification of SMA syndrome from medical records may also have resulted in underreporting of subtle or short-lasting manifestations, particularly mild language disturbances or motor initiation deficits. Although postoperative neurological deficits and SMA syndrome were recorded separately from KPS, KPS represents a global measure of functional status and lacks sensitivity for characterizing specific or subtle SMA-related motor and language deficits. Moreover, the relative contributions of neurological deficits, general postoperative recovery, frailty, and systemic complications to changes in KPS could not be systematically differentiated.

DTI-based tractography and connectivity analyses were not systematically available for the majority of patients. Consequently, the precise spatial relationship between the tumor and relevant functional cortical and subcortical pathways could not be systematically quantified or incorporated into the multivariable analyses, representing a potential source of residual confounding.

The single-center setting and moderate sample size may reduce generalizability and statistical power, as reflected by the wide confidence intervals in several regression analyses. The selection of covariates in the multivariable models was therefore guided by clinical relevance and established prognostic value rather than by data-driven variable selection. The mixed-grade composition of our cohort (CNS WHO grades 2–4) required application of two distinct RANO classification frameworks—CE-based for high-grade and T2-FLAIR-based for low-grade tumors—which limits direct comparison of resection extent across grade subgroups. Finally, detailed longitudinal assessment of recovery trajectories was beyond the scope of this study.

Despite these limitations, the present analysis provides consistent evidence that postoperative SMA syndrome does not adversely affect oncological management or survival outcomes in patients undergoing resection of SMA-associated gliomas.

## Conclusion

Postoperative SMA syndrome was not associated with delayed adjuvant therapy, reduced progression-free survival, or overall survival. Oncological outcomes were primarily determined by established prognostic factors, whereas postoperative functional decline influenced treatment intensity. These findings support maximal safe resection in the SMA region.

## Supplementary Information

Below is the link to the electronic supplementary material.

Supplementary Material 1

## Data Availability

No datasets were generated or analysed during the current study.
