# Supplementary material for: Supplementary motor area syndrome in intracranial gliomas is not associated with worse survival
Source: J Neurooncol. 2026 Sep 23;179(3):98. doi: 10.1007/s11060-026-05805-y (PMC13601069; doi:10.1007/s11060-026-05805-y)
Supplement: Supplementary file 1 [file 11060_2026_5805_MOESM1_ESM.docx]

**Supplementary Table S1.** Model-fit statistics for multivariable regression analyses

| **Analysis** | **Model** | **N** | **Events** | **Model-fit statistic** |
| --- | --- | --- | --- | --- |
| Postoperative SMA syndrome | Primary logistic model | 70 | 34 | Nagelkerke R² = 0.092 |
| Postoperative SMA syndrome | RANO sensitivity logistic model | 65 | 31 | Nagelkerke R² = 0.078 |
| Postoperative SMA syndrome | SMA-border sensitivity logistic model | 70 | 34 | Nagelkerke R² = 0.130 |
| Postoperative SMA syndrome | RANO class 2 vs class 1 adjusted logistic model | 54 | 26 | Nagelkerke R² = 0.181 |
| Postoperative SMA syndrome | RANO class 3 vs class 2 adjusted logistic model | 48 | 27 | Nagelkerke R² = 0.077 |
| Postoperative SMA syndrome | Resection limited by IONM adjusted logistic model | 70 | 34 | Nagelkerke R² = 0.095 |
| Postoperative SMA syndrome | Precentral gyrus involvement adjusted logistic model | 70 | 34 | Nagelkerke R² = 0.112 |
| Receipt of non-standard adjuvant therapy | Multivariable logistic model | 64 | 24 | Nagelkerke R² = 0.407 |
| Time to initiation of adjuvant therapy | Multivariable Cox model | 69 | 50 | C-index = 0.657 (95% CI 0.579–0.734) |
| Progression-free survival | Multivariable Cox model | 69 | 39 | C-index = 0.716 (95% CI 0.635–0.797) |
| Overall survival | Multivariable Cox model | 69 | 21 | C-index = 0.782 (95% CI 0.687–0.877) |
| Persistent SMA and time to adjuvant therapy | Multivariable Cox model | 34 | 24 | C-index = 0.662 (95% CI 0.559–0.766) |

Abbreviations: SMA, supplementary motor area; RANO, Response Assessment in Neuro-Oncology; IONM, intraoperative neuromonitoring; PFS, progression-free survival; OS, overall survival; CI, confidence interval. Model fit was assessed using Nagelkerke R² for logistic regression models and Harrell's concordance index (C-index) for Cox proportional hazards models.

**Supplementary Table S2.** Exploratory analysis for patients not recovered from SMA at first follow-up (median 43 days (IQR 15.5–97.5))

| **Variable** | **Recovered SMA syndrome (n=30)** | **Persistent SMA syndrome (n=7)** | **p-value** | **p-value** |
| --- | --- | --- | --- | --- |
| **Time to initiation of adjuvant therapy (median [IQR])** | 33.0 [21.2–79.8] | 21.0 [18.0–73.0] |  | **p-value** |
| **Patient survival,** univariate Cox regression analysis | | | **Adjusted HR (95% CI)** |  |
| **PFS, months (median [IQR])** | 15.1 [6.0–24.6] | 9.2 [6.5–16.7] | 0.67 (0.19–2.33), | 0.529 |
| **OS, months (median [IQR])** | 15.5 [10.2–32.3] | 11.4 [10.6–18.1] | 2.46 (0.73–8.26), | 0.145 |
| **Time to initiation of adjuvant therapy,** univariate Cox regression analysis | | |  |  |
| Persistent SMA deficits at follow-up |  |  | 0.91 (0.32–2.60), | 0.859 |
| Age at operation (per year) | 58.0 [31.5-65.0] | 61.0 [56.5-62.5] | 1.02 (1.00–1.05), | 0.103 |
| Preoperative KPS (per point) | 85.0 [70.0–90.0] | 70.0 [70.0-80.0] | 0.99 (0.97–1.02), | 0.585 |
| Tumour size (per cm³) | 25.6 [11.4-42.7] | 40.4 [26.0-45.6] | 1.01 (0.99–1.02), | 0.382 |

**Abbreviations:** SMA, supplementary motor area; KPS, Karnofsky Performance Status; HR, hazard ratio; CI, confidence interval; PFS, progression-free survival; OS, overall survival; IQR, interquartile range.

**Supplementary Table S2.** Model-fit statistics for multivariable regression analyses

| **Analysis** | **Model** | **N** | **Events** | **Model-fit statistic** |
| --- | --- | --- | --- | --- |
| Postoperative SMA syndrome | Primary logistic model | 70 | 34 | Nagelkerke R² = 0.092 |
| Postoperative SMA syndrome | RANO sensitivity logistic model | 65 | 31 | Nagelkerke R² = 0.078 |
| Postoperative SMA syndrome | SMA-border sensitivity logistic model | 70 | 34 | Nagelkerke R² = 0.130 |
| Postoperative SMA syndrome | RANO class 2 vs class 1 adjusted logistic model | 54 | 26 | Nagelkerke R² = 0.181 |
| Postoperative SMA syndrome | RANO class 3 vs class 2 adjusted logistic model | 48 | 27 | Nagelkerke R² = 0.077 |
| Postoperative SMA syndrome | Resection limited by IONM adjusted logistic model | 70 | 34 | Nagelkerke R² = 0.095 |
| Postoperative SMA syndrome | Precentral gyrus involvement adjusted logistic model | 70 | 34 | Nagelkerke R² = 0.112 |
| Receipt of non-standard adjuvant therapy | Multivariable logistic model | 64 | 24 | Nagelkerke R² = 0.407 |
| Time to initiation of adjuvant therapy | Multivariable Cox model | 69 | 50 | C-index = 0.657 (95% CI 0.579–0.734) |
| Progression-free survival | Multivariable Cox model | 69 | 39 | C-index = 0.716 (95% CI 0.635–0.797) |
| Overall survival | Multivariable Cox model | 69 | 21 | C-index = 0.782 (95% CI 0.687–0.877) |
| Persistent SMA and time to adjuvant therapy | Multivariable Cox model | 34 | 24 | C-index = 0.662 (95% CI 0.559–0.766) |

Abbreviations: SMA, supplementary motor area; RANO, Response Assessment in Neuro-Oncology; IONM, intraoperative neuromonitoring; PFS, progression-free survival; OS, overall survival; CI, confidence interval. Model fit was assessed using Nagelkerke R² for logistic regression models and Harrell's concordance index (C-index) for Cox proportional hazards models.
